# Real-Time Electrical Impedimetric Monitoring of Blood Coagulation Process under Temperature and Hematocrit Variations Conducted in a Microfluidic Chip

**DOI:** 10.1371/journal.pone.0076243

**Published:** 2013-10-07

**Authors:** Kin Fong Lei, Kuan-Hao Chen, Po-Hsiang Tsui, Ngan-Ming Tsang

**Affiliations:** 1 Graduate Institute of Medical Mechatronics, Chang Gung University, Taoyuan, Taiwan, Republic of China; 2 Department of Mechanical Engineering, Chang Gung University, Taoyuan, Taiwan, Republic of China; 3 Department of Medical Imaging and Radiological Sciences, Chang Gung University, Taoyuan, Taiwan, Republic of China; 4 School of Traditional Chinese Medicine, Chang Gung University, Taoyuan, Taiwan, Republic of China; 5 Department of Radiation Oncology, Chang Gung Memorial Hospital, Taoyuan, Taiwan, Republic of China; Emory University/Georgia Insititute of Technology, United States of America

## Abstract

Blood coagulation is an extremely complicated and dynamic physiological process. Monitoring of blood coagulation is essential to predict the risk of hemorrhage and thrombosis during cardiac surgical procedures. In this study, a high throughput microfluidic chip has been developed for the investigation of the blood coagulation process under temperature and hematocrit variations. Electrical impedance of the whole blood was continuously recorded by on-chip electrodes in contact with the blood sample during coagulation. Analysis of the impedance change of the blood was conducted to investigate the characteristics of blood coagulation process and the starting time of blood coagulation was defined. The study of blood coagulation time under temperature and hematocrit variations was shown a good agreement with results in the previous clinical reports. The electrical impedance measurement for the definition of blood coagulation process provides a fast and easy measurement technique. The microfluidic chip was shown to be a sensitive and promising device for monitoring blood coagulation process even in a variety of conditions. It is found valuable for the development of point-of-care coagulation testing devices that utilizes whole blood sample in microliter quantity.

## Introduction

Blood coagulation is an extremely complicated and dynamic physiological process. It provides an important mechanism for maintaining hemostasis [1]. However, disorders in blood coagulation may lead to serious pathological complications, such as hemorrhage, thrombosis, and embolism in the vascular system. In cardiac surgical procedures, monitoring of blood coagulation is essential to predict the risk of hemorrhage and thrombosis. Continuous measurement of the blood coagulation process can ensure that the patient is effectively treated with an anticoagulant to avoid blood clots.

Currently, the activated partial thromboplastin time (APTT) test is the standard test used in hospital to be a performance indicator measuring the efficacy of coagulation pathways [2], [3]. Apart from detecting abnormalities in blood clotting, it is also used to monitor the treatment effects with anticoagulant. In APTT test, blood samples are collected in tubes with oxalate or citrate to prevent coagulation by binding calcium. The plasma sample is then separated from whole blood by centrifugation in the laboratory. It is mixed with reagents including phospholipids, activator (such as silica, celite, kaolin, or ellagic acid), and calcium in order to activate the intrinsic pathway of blood coagulation. The mixture is observed for clotting and the clotting time is recorded. Fibrin formation in blood is a result of a complex cascade of catalytic reactions which involve multiple coagulation factors. A number of methods have been reported to detect the fibrin formation in blood, such as optical measurement [4], ultrasonic measurement [5], and sensing the change in the mechanical [6], [7] and electrical [8]–[10] properties of blood. These methods are required to operate with bulky equipment in laboratory. Moreover, the acquirement of plasma in APTT test is time consuming and labor intensive. The storage conditions of blood need to be constant and any deviations could cause changes in properties such as pH of blood plasma. Hence, the APTT test result might not reflect the true condition of the patients. Therefore, the need for a point-of-care coagulation testing device that utilizes whole blood samples in microliter quantities is emphasized recently.

Among the aforementioned detection methods, it was reported that electrical impedance measurement is the best method for whole blood coagulation time measurement [11]. Moreover, it is an appropriate method for the development of point-of-care coagulation testing device because the electric signal of the measurement output can be analyzed by compact electrical circuit [12]. From literature, magnitude of the impedance change correlates to the fibrinogen concentration of the plasma [8]–[10]. The impedance of blood was found to represent erythrocyte sedimentation rates (ESR) [13] and hematocrit [14], [15], and evaluate the quality of stored blood [16]. The electrical impedance method was reported to be reproducible and exhibit good correlation with other standard detection methods such as optical absorbance. Alternatively, miniaturization and disposability are the other important requirements for the development of point-of-care coagulation testing device for routine tests [17]. Microfluidic technology provides a technique to miniaturize the electrical impedance measurement device for blood coagulation test.

In the past two decades, microfluidic system has been rapidly developed from early single channel device [18] to current complex analysis system [19]. Due to the mature development of the micro-fabrication technology, various biomedical applications have been demonstrated, such as immunoassay [20]–[24], DNA-based analysis [24]–[26], and cellular analysis [27]–[29]. Point-of-care biomedical devices for emergency or home use can be realized due to their advantages of miniaturization, integration, and automation of biological and chemical testing. One of the objectives of the development of microfluidic systems is to implement the entire conventional laboratory protocols automatically. The bioanalytical process can be carried in a well-controlled and precise environment including sample/reagent volume, flow velocity, temperature, and incubation time. Therefore, microfluidic system is often interpreted to a miniaturized version of a conventional laboratory and therefore it can provide a precise and well-controlled environment for the investigation of the blood coagulation process. Recently, blood coagulation testing has been also implemented by microfluidic systems. A system with thermoexpandable polymer actuator was developed for the actuation of biological fluid in a microchannel [30]. Fluid progression in the channel can be monitored optically and the flow rate of 10–60 nl/min was achieved. During the blood coagulation test, blood was injected to the system and its progression can be monitored. Blood coagulation characterization was performed based on an increase in blood viscosity. Similar measurement method was utilized for the monitoring of blood coagulation [31]. A thermopneumatic microfluidic actuator was used to manipulate the blood sample in the microchannel. Obstruction of the microchannel by clotted blood was observed and the position of the blood plug determined the blood coagulation time. The above demonstrations were based on visual observation to determine the blood coagulation in microfluidic systems and quantitative results could be not achieved. Hence, a microfluidic chip has been fabricated and APTT test was performed using whole blood samples by detecting the change in electrical impedance of blood during coagulation [32]. Quantitative determination of blood coagulation time was demonstrated. Monitoring of blood coagulation is essential to predict the risk of hemorrhage and thrombosis in surgical procedures. But, blood coagulation time is influenced by variation of conditions, i.e., temperature and hematocrit. For example, hypothermia is associated with an increased risk of hemorrhage and blood in a high hematocrit is in a high risk of thrombosis [33]–[35]. However, investigation of blood coagulation process using impedance measurement under temperature and hematocrit variations has not yet been explored.

A device for the impedimetric determination of blood coagulation process has been developed with the advantages of miniaturization and simple operation. Blood coagulation time under temperature and hematocrit variations can be determined by the impedance measurement technique. Real-time quantitative determination of blood coagulation process can be realized and this technique provides a promising tool to predict the risk of hemorrhage and thrombosis in surgical procedures. In this work, a high throughput microfluidic chip is fabricated for the study of the blood coagulation time under temperature and hematocrit variations. The chip consists of 7 identical measurement wells, in which a pair of electrodes is located at the bottom of each well. Microliter volume of whole blood samples can be respectively loaded into the wells and simultaneous multiple analyses are realized for routine coagulation tests. During coagulation, magnitude and phase angle of electrical impedance of the blood were recorded by the electrodes in contact with the blood. Electrical impedance of whole blood was found to be increasing until coagulation. Analysis of the impedance change of blood before and after coagulation was conducted to investigate the characteristics of blood coagulation process and the starting time of blood coagulation was defined. Blood coagulation time under temperature and hematocrit variations was then studied to investigate the sensitivity of such detection technique.

## Materials and Methods

### Design and Fabrication of the Microfluidic Chip

**Figure 1:**
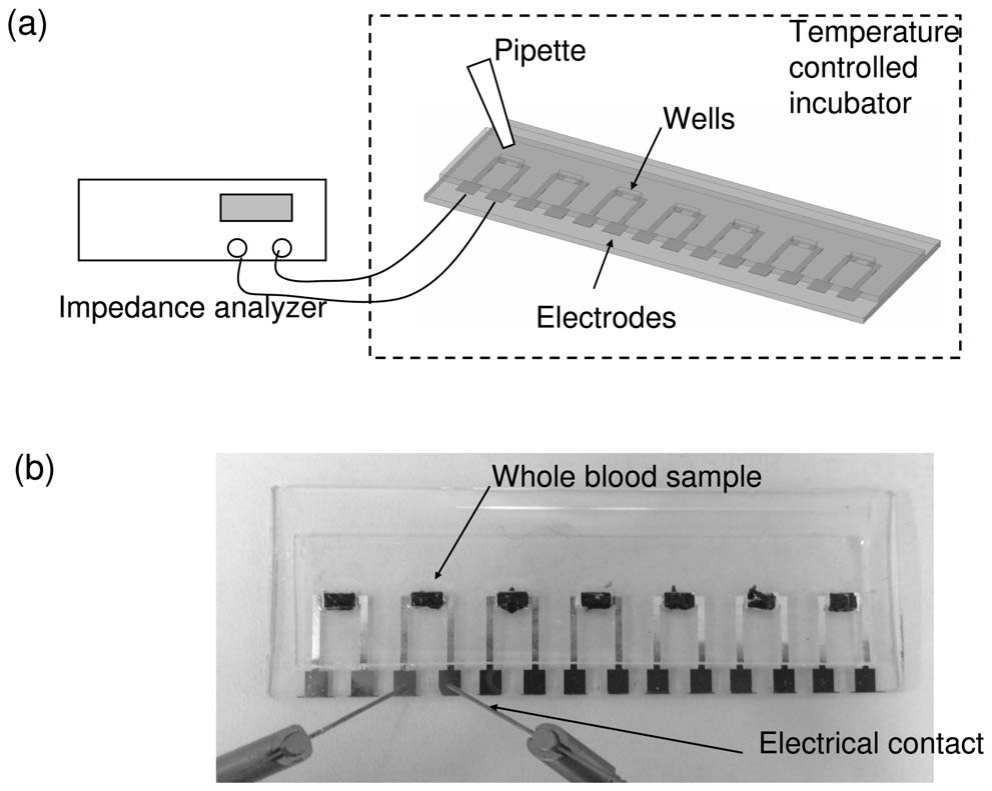
Microfluidic chip for real-time electrical impedimetric monitoring of blood coagulation process. (a) Illustration of the experimental setup. (b) Photograph of the microfluidic chip. The blood samples were loaded to the measurement wells respectively.

A high throughput microfluidic chip is fabricated and provides a precise and well-controlled environment for the investigation of blood coagulation under various conditions. The chip is a glass substrate with 7 pairs of aluminum electrodes and is covered with a 1 mm thick polydimethylsiloxane (PDMS) layer with 7 corresponding measurement wells. The electrodes are utilized for the measurement of the electrical impedance of the blood sample and the wells are used for containing blood samples. The distance of each pair of electrodes is 1.8 mm and the dimension of each well is 2×1.8×1 mm^3^. The blood sample (10 µl) is loaded to the well quantitatively by manual pipetting. By using the microfluidic fabrication technology, the dimension of the wells and the electrodes and the volume of blood sample can be well controlled for a precise study of the blood coagulation process. Hence, the chip is placed into a temperature controlled incubator for the study of the blood coagulation process. Electrical impedance of the whole blood sample is recorded by an impedance analyzer (VersaSTAT 4, Ametek, USA) across the electrodes in contact with the blood samples. Illustration of the experimental setup and photograph of the microfluidic chip are shown in Figure 1.

**Figure 2:**
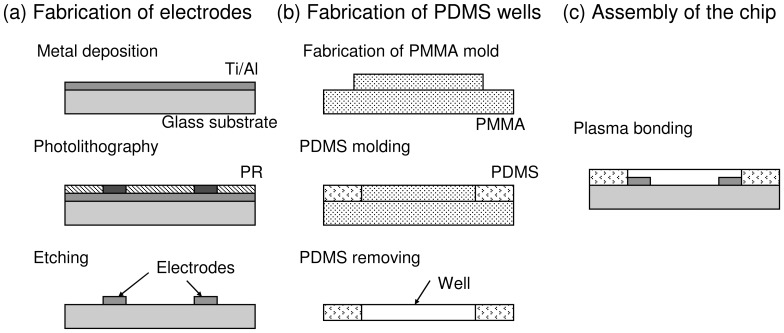
Fabrication process of the microfluidic chip. (a) Fabrication of the electrodes on glass substrate. (b) Fabrication of the PDMS wells. (c) Assembly of the microfluidic chip.

The microfluidic chip is a glass substrate with 7 pairs of electrodes and is covered with a PDMS (Sylgard^®^ 184, Dow corning, USA) layer with 7 corresponding measurement wells. Micro-fabrication technique is involved for the fabrication of the microfluidic chip and illustrated in Figure 2. Briefly, Ti/Al electrodes were first fabricated on the glass substrate by metal deposition, photolithography, and metal etching process, respectively. The PDMS layer with wells was then fabricated by soft lithography. The general process of soft lithography is briefly described. PDMS mixture is first prepared by thoroughly mixing of the PDMS pre-polymer and curing agent in a weight ratio of 10∶1 according to the manufacturer’s instruction. Then, the mixture is degassed under a vacuum chamber, and followed by pouring onto a polymethylmethacrylate (PMMA) mold with designed structure fabricated by micro-machining technique. After curing at 70°C for 1 h, the PDMS layer with the desirable structures is obtained through a careful de-molding process. Finally, the microfluidic chip was fabricated by the bonding of the glass substrate and PDMS layer using oxygen plasma treatment.

### Blood Sample Handling

The blood sample was porcine blood that was collected from local slaughterhouse (Chung Mei Ham Food, Taiwan). Permission of the blood sample for the experimental usage was obtained from the slaughterhouse. Fresh whole blood was mixed with 15% acid citrate dextrose (ACD) anticoagulation solution at the slaughterhouse in order to prevent coagulation during transportation. Then, the blood was passed through a sponge to filter out impurities, such as hair and fatty tissue, and discarded whenever coagulation is observed. The whole blood was subsequently centrifuged at 3,200 rpm for 12 min to separate the blood into the composition of packed erythrocytes and the plasma. The red blood cells were then rinsed twice by a buffered saline solution. Then, the erythrocytes and the plasma were stored at 4°C before the experiments. Blood sample in storage was discarded if the experiments were not started within 1 week.

### Electrical Impedance Measurement of the Blood Sample under Temperature and Hematocrit Variations

The red blood cells aggregation is a normal, reversible, physiological process occurring in whole blood. It is the kinetics of rouleaux formation depending on the various parameters, such as temperature and hematocrit. The aggregation may increase blood viscosity, increase flow resistance, form sludge blood in vessels, increase the interaction of leukocytes with the endothelium, and promote blood coagulation. As the coagulation mechanism is induced, thrombin acts as an enzyme to convert fibrinogen into fibrin fibers that enmesh blood cell and plasma. The blood then becomes a solid gel and a clot forms that comprises a meshwork of fibrin fibers running in all directions that entrap erythrocytes and plasma. The coagulation of blood is an extremely complicated and dynamic process and this work is to investigate the blood coagulation process based on the electrical impedance change of the blood.

Prior to each measurement, the erythrocytes and the plasma were taken out from the storage and placed at room temperature for 30 min. Then, the whole blood sample was mixed by the erythrocytes and the plasma at a certain volume ratio. The ratio is represented by hematocrit. Blood coagulation is induced by adding 0.5 M CaCl_2_ solution into the blood sample in the volume ratio of 1∶10. Hence, 10 µl blood sample was loaded to the well on the microfluidic chip by manual pipetting. The chip was placed in a temperature controlled incubator and investigation of the blood coagulation process was started. Electrical impedance of the blood including magnitude and phase angle was measured across the electrodes. Potential of 0.1 V was applied and the impedance was measured from 100 Hz to 10 kHz. The impedance of blood was recorded continuously with a step of 20 sec until the finish of blood coagulation.

**Figure 3:**
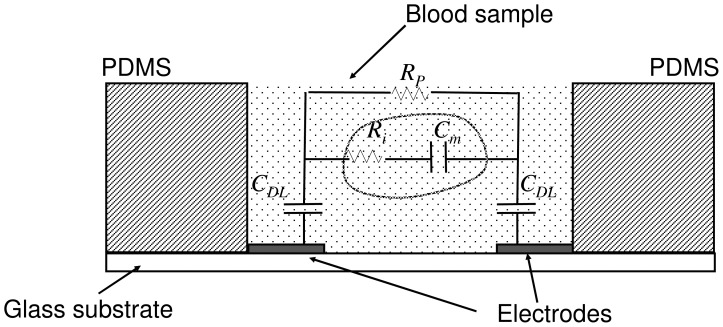
Equivalent electrical model of the whole blood. *R_p_* represents the plasma resistance. *R_i_* represents the red blood cell interior resistance. *C_m_* represents the red blood cell membrane capacitance. *C_DL_* presents the double layer capacitance between the electrode and the electrolyte.

The equivalent electrical model of the whole blood has been described in previous reports [36], [37]. The electrical impedance of blood can be closely simulated by a simple circuit consisting of plasma resistance *R_p_* connected to parallel with cell interior resistance *R_i_* and cell membrane capacitance *C_m_* in series. These parameters were assumed to be aggregated numbers. In our study, since a pair of planar electrodes was used for the measurement, the entire model was modified and a pair of double layer capacitances *C_DL_* was introduced. The equivalent electrical model is shown in Figure 3. In this circuit diagram, the total impedance was suggested to be interpreted as having two parallel branches. One branch represented the plasma resistance and another branch represented the impedance of the red blood cells.

## Results and Discussion

### Characterization of the Blood Coagulation Process

**Figure 4:**
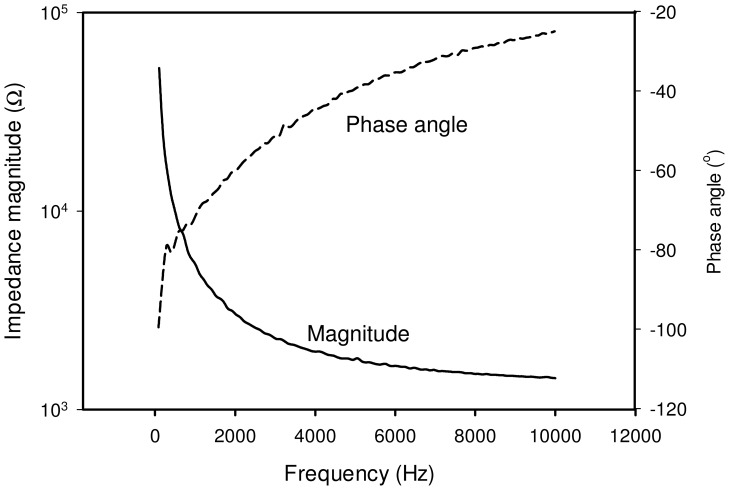
Impedance spectrum (magnitude and phase angle) of the whole blood between 100 Hz to 10 kHz. Blood sample in hematocrit of 45% was measured at 37°C. The solid line represents the impedance magnitude and the dash line represents the phase angle.

**Figure 5:**
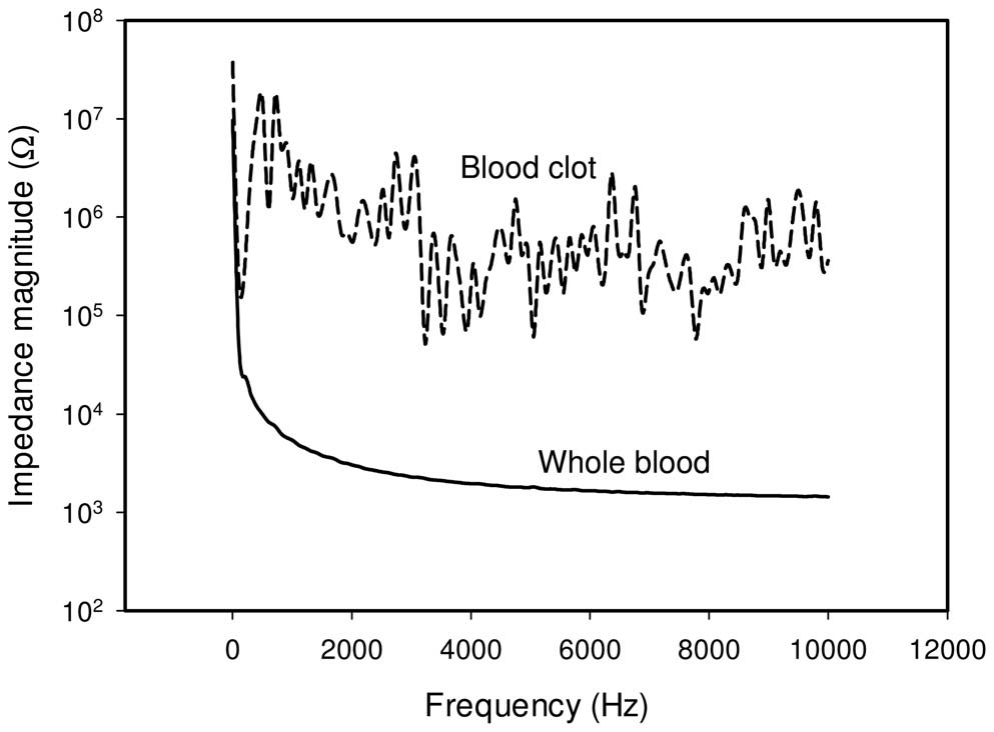
Impedance spectrum of the whole blood and the blood clot. The blood clot shows insulated, providing the impedance is high and the spectrum is fluctuated. Blood sample in hematocrit of 45% was measured at 37°C. The solid line and dash line represent the impedance spectrum of the whole blood and the blood clot, respectively.

The impedance spectrum of the whole blood was first studied using the microfluidic chip. Blood sample in hematocrit of 45% was loaded into the well and the chip was placed in the incubator at 37°C for the measurement. The spectrum of the whole blood is shown in Figure 4 and represents a general capacitive characteristic. At very low frequency, i.e., below 500 Hz, the magnitude is relatively high and the phase angle is around −90°. In contrast, at frequency above 500 Hz, the effect of capacitance is diminished. The magnitude and phase angle are observed to become relatively low and small gradually. The impedance spectrum matches the equivalent electrical model shown in Figure 3. That is, at frequency below 500 Hz, the impedance is dominated by the combination of the cell membrane capacitance *C_m_* and the double layer capacitance *C_DL_*. At frequency above 500 Hz, the cell interior resistance *R_i_* and plasma resistance *R_p_* become significant. When the blood is clotted, it becomes a solid gel that comprises a meshwork of fibrin fibers running in all directions that entrap erythrocytes and plasma. The electrical property of the blood clot shows insulated, providing that the impedance is high, i.e., ∼1 MΩ, and the impedance spectrum is fluctuated. The impedance spectrum of the whole blood and the blood clot is shown in Figure 5. In this work, the equivalent electrical model is similar to most of the electrical impedance measurements of cellular monitoring [27]–[29]. In the previous studies, cells laid on the electrode surface or suspended in the culture medium in the microfluidic chamber. When a potential was applied across the electrodes, cells blocked the current flow. The electrical model is simulated as having two parallel pathways, i.e., impedance contributed from cells and impedance contributed from medium. Therefore, the change of cell number induced the impedance change across the electrodes. In our study, when the blood is clotted, the plasma changes from conductive liquid form to insulated solid gel form. The plasma resistance dominates the impedance magnitude and the impedance change between the whole blood and the blood clot is significant.

**Figure 6:**
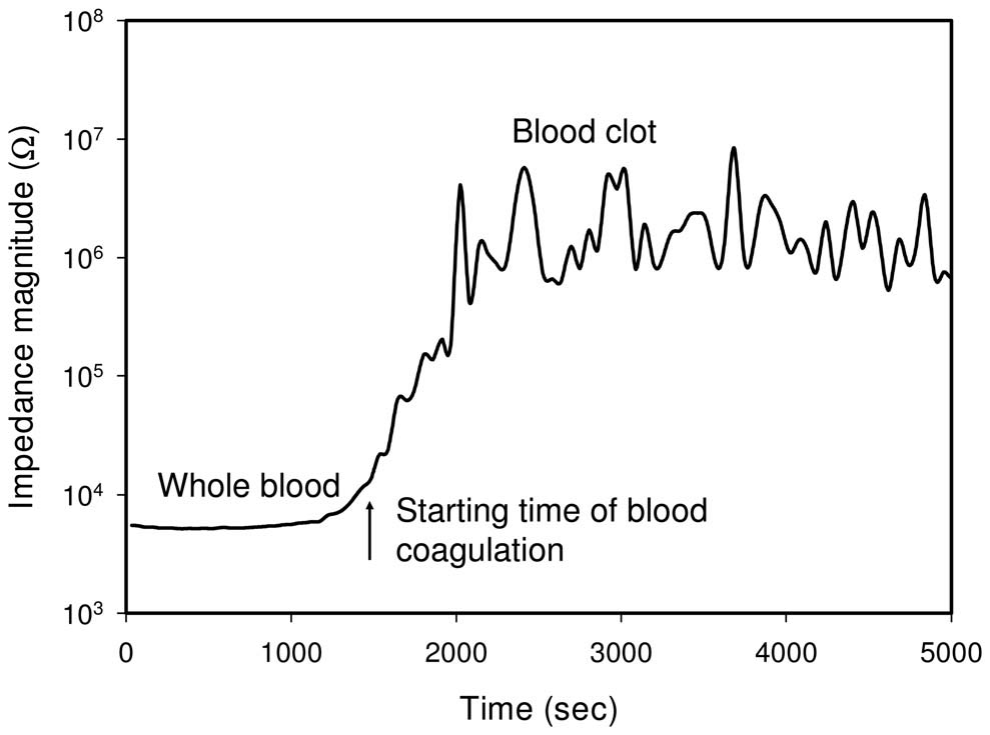
Blood coagulation process represented by the impedance magnitude at 1000 Hz. The starting time of the blood coagulation is defined at the impedance magnitude which is 2 times the magnitude of the whole blood. Blood sample in hematocrit of 45% was measured at 37°C.

Blood coagulation is an extremely complicated and dynamic physiological process and is triggered by the rupturing of endothelium due to external or internal injury. Since serious hemorrhage can be life threatening, efficient hemostasis is an important mechanism. When the blood is exposed to extra vascular tissue, clotting factors such as blood platelets and plasma protein fibrinogen are initiated immediately. Platelets form a plug at the site of injury and occlude the vascular lesion. Simultaneously, coagulation cascade begins to form fibrin strands, which can strengthen the platelet plug. Fibrin formation is a result of a complex cascade of catalytic reactions which involve multiple coagulation factors [38]–[39]. The cascade consists of intrinsic (contact activation) pathway and extrinsic (tissue factor) pathway and is rapid. Both pathways are interrelated and function simultaneously during the process of mammalian homeostasis. As a result of fibrin mesh formation, blood is considered to have clotted. Meanwhile, anticoagulant mechanisms ensure control of this coagulation process and prevent thrombotic diseases. In the study of blood coagulation process, impedance magnitude at 1000 Hz is measured with time and shown in Figure 6. Since the impedance magnitude at 1000 Hz of the blood is composed of the impedance contributed from plasma and cells, it is defined to represent the blood coagulation status for the investigation of the blood coagulation process. Result reveals the rapid formation of fibrin during the coagulation cascade, which can be monitored by the impedance change. Moreover, the starting time of the blood coagulation is defined at the impedance magnitude which is 2 times the impedance of the whole blood.

### Blood Coagulation Time under Temperature Variations

**Figure 7:**
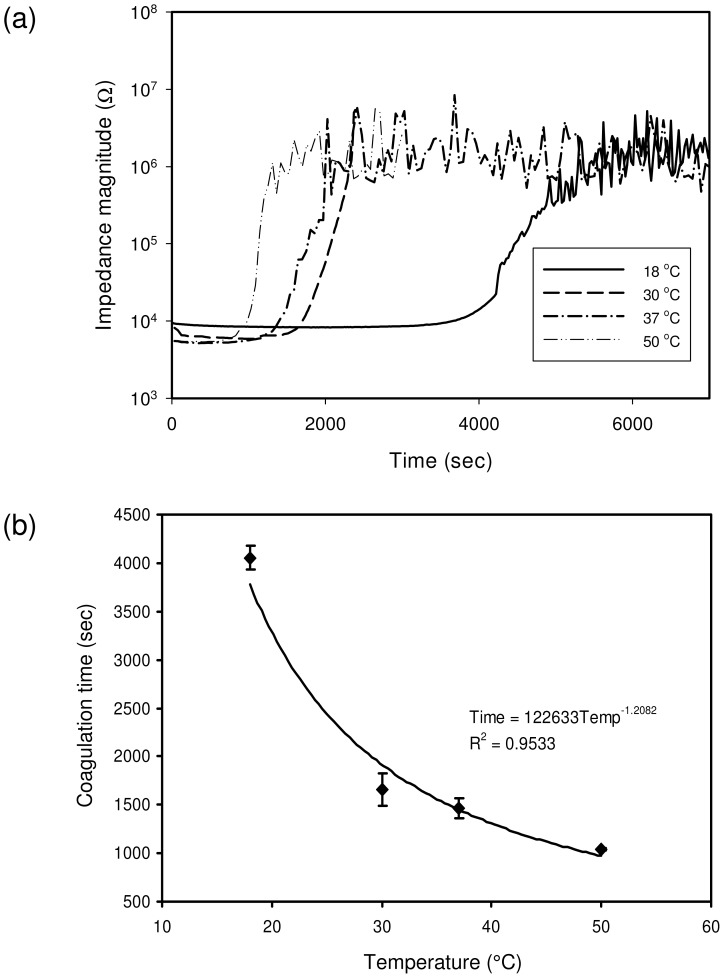
Blood coagulation time under temperature variations of 18, 30, 37, and 50°C. (a) Blood coagulation response under temperature variations. (b) Correlation between coagulation time and temperature. The impedance magnitude of the blood sample was measured at 1000 Hz. Blood sample in hematocrit of 45% was used. Error bars represent the standard deviations of three repeated measurements.

In most of the biological reactions, temperature variation is an important issue to influence the completion of the process. Similarly, blood coagulation has been reported to be influenced by the temperature clinically [33]. Enzyme activities and platelet activation were only slightly reduced at 33°C compared with 37°C. However, they were significantly reduced below 33°C. As a result, longer blood coagulation time is resulted and significantly increased risk of death is associated after trauma. Hypothermia is associated with an increased risk of hemorrhage during surgical procedures. In this study, blood coagulation time was investigated using impedance measurement under temperature variations of 18, 30, 37, and 50°C. Blood sample in hematocrit of 45% was used and loaded into the well in the microfluidic chip. The chip was placed in the incubator and the measurements were performed at different temperatures respectively, as shown in Figure 7(a). Generally, the blood coagulation time, which was defined as the starting time of the blood coagulation process, was shown to be shortened with the increase of the temperature. In order to have better observation, the correlation between the coagulation time and temperature was plotted and is shown in Figure 7(b). It was shown that the correlation did not have a linear relationship, but power relationship. The blood coagulation time at 18°C was much longer than the others, which is in a good agreement with results in the previous clinical report [33]. The activity of the coagulation factors was reduced much at lower temperature then 37°C. Therefore, longer time for fibrin formation was taken. Moreover, blood coagulation time at 50°C was slightly shortened than the time at 37°C. That indicated the activity of the coagulation factors was not significantly enhanced at higher temperature then 37°C. The impedance measurement technique was shown sensitive to the blood coagulation time under temperature variations. Three repeated measurements were conducted and the R-squared value was 0.9533.

### Blood Coagulation Time under Hematocrit Variations

**Figure 8:**
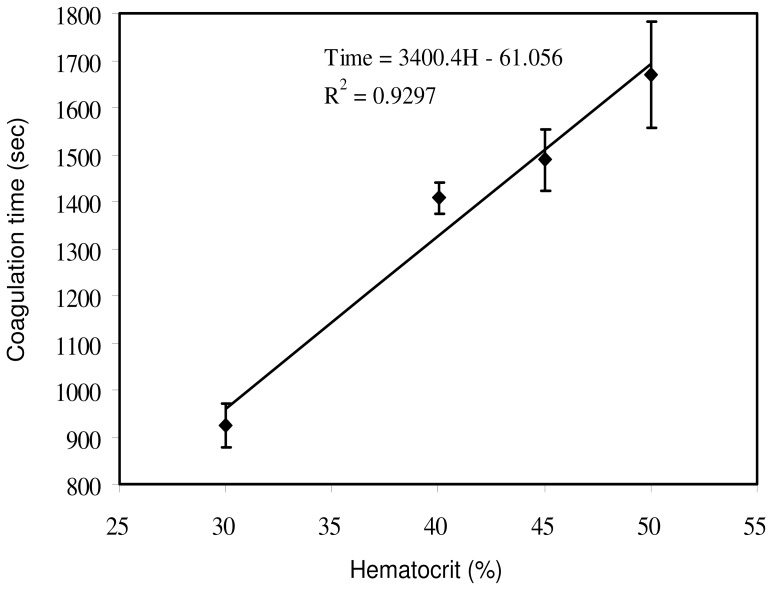
Correlation between blood coagulation time and hematocrit of 30, 40, 45, and 50%. The impedance magnitude of the blood sample was measured at 1000 Hz and 37°C. Error bars represent the standard deviations of three repeated measurements.

The fibrinogen in the plasma plays an important role in the blood coagulation process. Hematocrit represents the volume percentage of red blood cells in blood. Normally, it is about 45% for men and 42% for women [40]. For the blood sample at a low hematocrit, there could be more fibrinogen able to be converted into fibrin fibers than that of blood at a high hematocrit. Consequently, a linear proportional relationship was shown between the blood coagulation time and the hematocrit in the blood sample based on ultrasonic measurement [5], . Clinically, a high hematocrit is expected to be associated with an increased risk of thrombosis or embolism [34], [35]. An effective technique for the measurement of hematocrit in blood sample is important. In this study, blood coagulation time of the blood sample in various hematocrit values, i.e., 30, 40, 45, and 50%, was measured at 37°C using impedance measurement technique. The correlation between the blood coagulation time and hematocrit was plotted and is shown in Figure 8. Results validated that the higher hematocrit of the blood sample, the longer period of duration is needed for clotting. Linear relationship was observed in the current investigation and agreed with the previous studies [5], [41]. Three repeated measurements were conducted and the R-squared value was 0.9297. Real-time quantitative description of the blood coagulation process under temperature and hematocrit variations can be realized by the proposed technique. It showed a promising tool for the real-time determination of the blood coagulation process.

### Conclusion

A high throughput microfluidic chip has been developed and provides a precise and well-controlled environment for the investigation of the blood coagulation process. Blood coagulation time was determined by the electrical impedance of the blood sample, which showed a promising technique to quantify the blood coagulation process. Since a small volume, i.e., microliter, of the blood sample was utilized in the microfluidic chip, homogeneity of the sample can be ensured. In this work, analysis of the impedance change of blood sample during coagulation was conducted and the starting time of blood coagulation was defined by the impedance magnitude. Moreover, blood coagulation time under temperature and hematocrit variations was studied and found a good agreement with results in the previous clinical report. Results showed that the impedance measurement using the microfluidic chip was a sensitive and promising technique for monitoring blood coagulation process even in variation of conditions. Electrical impedance determination of blood coagulation time provides a fast and easy measurement compared with the conventional analysis techniques. This work is found valuable for the development of clinical equipment for routine coagulation tests.
